# Techno-Economic Analysis of Biofuel Production from Macroalgae (Seaweed)

**DOI:** 10.3390/bioengineering4040092

**Published:** 2017-11-26

**Authors:** Mohsen Soleymani, Kurt A. Rosentrater

**Affiliations:** 1 Department of Biosystems Engineering, Shahid Chamran University of Ahvaz, Ahvaz 61357-8315, Iran; mohsensoleymanig@gmail.com; 2 Department of Agricultural and Biosystems Engineering, Iowa State University, 3327 Elings Hall, Ames, IA 50011, USA

**Keywords:** techno-economic analysis, seaweed, breakeven selling price, sustainable energy

## Abstract

A techno-economic evaluation of bioenergy production from macroalgae was carried out in this study. Six different scenarios were examined for the production of different energy products and by-products. Seaweed was produced either via the longline method or the grid method. Final products of these scenarios were either ethanol from fermentation, or electricity from anaerobic digestion (AD). By-products were digestate for AD, and animal feed, or electricity and digestate, for the fermentation pathway. Bioenergy breakeven selling prices were investigated according to the cost components and the feedstock supply chain, while suggestions for potential optimization of costs were provided. The lowest production level of dry seaweed to meet 0.93 ($/L) for ethanol fuel and 0.07 $/kW-h for electricity was found to be 0.68 and 3.7 million tonnes (dry basis), respectively. At the moment, biofuel production from seaweed has been determined not to be economically feasible, but achieving economic production may be possible by lowering production costs and increasing the area under cultivation.

## 1. Introduction

Since 1900 the amount of global carbon emissions has increased, due to increasing use of fossil fuels for transportation, industry, and private enterprises. Additionally, the rate of emissions has increased in recent decades; emissions increased by over 16 times between 1900 and 2008, and about 1.5 times during 1990–2008 alone [1]. Environmental challenges of fossil fuel use, along with other issues, such as dynamic swings in crude oil prices and challenges in energy security, to name a few, have made the replacement of these environmentally harmful and unsustainable fuels by renewable and sustainable alternatives necessary [2,3]. Bioethanol, which is considered a renewable energy source, potentially can reduce transportation emissions in addition to replacing a portion of the petroleum-based fuel supply [4,5], even though its current production is not enough to meet all of current fuel demand. The present substrates for bioethanol production (predominantly corn and sugarcane) compete directly with human foods by using arable lands, water, fertilizer, and other resources, and arguably may have negative effects on food prices [5,6]. Therefore, much attention is now focused on producing biofuels from lignocellulosic biomass, agricultural wastes, and other biological materials. Although these feedstocks do not compete directly with human food resources, they can compete indirectly if they are cultivated in available arable lands [7]. Also because cellulosic biomass has high lignin content, its conversion into biofuels can be difficult and cost-prohibitive [5]. 

Since algae grow in marine waters [5,8], algal biofuels, which are considered “third-generation biofuels” [2], may help change the food vs. fuel argument. Yes, it is true that large-scale adoption of this approach can potentially have negative effects, but it can allow highly productive land to be used for food production as opposed to crops for biofuels. Macroalgae, or seaweed, has no lignin but high moisture (70–90%) and ash (21.5–33.4%) levels [3,9]. Low lignin in macroalgae makes it well matched to biogas production in anaerobic digesters [10]. On the other hand, easily fermentable carbohydrates, including laminarin and mannitol, especially in brown macroalgae, are suitable for bioethanol conversion [5,8,11]. 

In spite of valuable food and medical products [12,13], which are produced from seaweeds, their profitability as energy crops has not yet been commercially confirmed. Seaweed cultivation can be very labor intensive, and also can require expensive equipment [5,14]. The potential profit of the seaweed-based renewable energy industry will hopefully be high enough to offset these high costs [15]. It may be possible to achieve this level of profitability, but only by increasing the efficiency and scale of current production [8]. 

Cultivation costs will vary according to the geographical origin, cultivation methods, cultivation scale, yield per unit area, technologies used, transportation methods, and various pretreatment operations [8,10,16,17,18]. For example, the net profit for a farmer with a four-person family in 2012 in Mexico and Indonesia was only just higher than the International Poverty Line [18]. [19] suggested that to have a profitable seaweed farm, the products should be sold at higher price (for example €2/kg wet basis), or the farm should be expanded by production of other valuable products (e.g., scallops) in order to have supplemental income.

[8] conducted a study to compare the cost of production of ethanol, methane (then converted to gasoline via syngas and methanol), and biodiesel derived from seaweed. This study found that fuel production from seaweed was not economically feasible unless you considered the production of valuable by-products such as alginates, mannitol, and iodine, which could help offset the production costs.

The breakeven selling price for electricity generated from seaweed has been estimated at around €120/MW-h ($154 if €1 = $1.28) [10]. This price may be acceptable compared with some other renewable energy prices, such as solar thermal ($251/MW-h), solar photovoltaic (157 $/MW-h), and biomass-generated electricity (120.2 $/MW-h) [20]. 

Nonetheless, economic studies of biofuel production from seaweed are few in number. Several, including [21], have however investigated the costs to produce seaweed. Published papers which have examined the use of seaweed to produce biofuels and/or bioenergy include [22] as well as [23]. Relatedly, [24] assessed the costs for production of biofuels from microalgae, not seaweed.

Consequently, due to the dearth of published studies, the economic investigation of this emerging energy resource is necessary. Thus the aim of this study was to investigate different methods of seaweed cultivation and conversion into bioenergy, to determine the most economical combination of these methods, and to determine the minimum scale of economical seaweed cultivation.

## 2. Methods

### 2.1. Data Sources, Calculation Methods, Scenarios, and Cost Analysis

All economic analyses in this study were conducted using US dollars ($). Any economic data found in other currencies (i.e., Euros) were converted prior to analyses.

**Figure 1:**
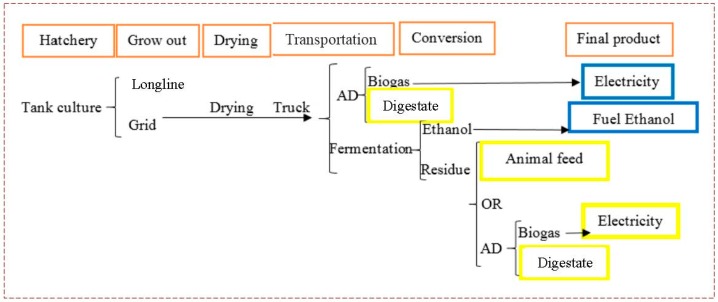
Overview of the supply chain and biofuel production methods. Orange rectangles indicate the main steps, blue rectangles indicate the final products in each scenario, and yellow indicate the by-products in each scenario. The red dashed box indicates the system boundary.

Six different scenarios (Table 1) were simulated for the production of different energy products and by-products. The system boundary of the production system that we analyzed is illustrated in Figure 1. Seaweed was produced either via the longline method or the grid method. Final products of these scenarios were either ethanol from fermentation, or electricity from anaerobic digestion (AD) (which could be followed by an integrated Combined Heat and Power (CHP) system). By-products were digestate for AD, and animal feed, or electricity and digestate, for fermentation. Figure 1 illustrates these scenarios. 

This system begins with seaweed production, including hatchery and grow out farms. Longlines and continuous culture grid units were the two methods considered for seaweed biomass production in a typical offshore farm. Mature seaweeds, after the growing season, are harvested by boats and transported by barges or boats to the shoreline. To have a continuous supply in the industrial portion of the supply chain, seaweeds should be shelf stable for a long potential storage time. Therefore, to prevent spoilage and assure an appropriate shelf life, harvested seaweed must be dried to under 10% moisture content. For many food and feed products, recommended moistures are less than 10%, in fact. Moreover, dry seaweed requires lower space and fuel consumption for transport than wet seaweed. In this study, it is assumed that all land transportation is carried out by trucks. 

To conduct a comprehensive techno-economic analysis, all capital and operational costs were determined at multiple production scales (0–2 million dry tonnes of seaweed per annum) for the supply chain illustrated in Figure 1. All equipment and operational data were taken from published literature. The lifetime of all equipment was considered to be 10 years. Equipment costs were assumed to be constant worldwide. The based currencies were converted to US dollars, based on average conversion rates in the original year and then all costs were converted to US dollar in 2013 according to the inflation rate between the original year and 2013. The original costs of small capacity equipment and industries in literature were converted to costs of 95 ML capacity, using a scaling equation (Equation (1)) [25]:

(1) $$\mathrm{New}\;\cos t=Original\;cost\;{\left(\frac{New\;size\left(capacity\right)}{Original\;size\;\left(capacity\right)}\right)}^{0.6}$$

Annual fuel ethanol production rate was considered to be 95 ML (95% ethanol and 5% gasoline, volumetrically) based on [25]. The annual requirement of fresh and dry seaweed production was calculated according to the ethanol production rate (75 kg ethanol per 1 ton dry seaweed, [4], and moisture content of fresh seaweed (85%, mass based on [19]). Energy (electricity, heat, and fuel) costs were based on USA average prices in 2013. Labor cost was considered according to the average labor earnings in USA in 2013. 

Marketing prices for animal feed and digestate were considered to be 590 and 9.75 $/t [10], respectively. Also, the marketing price of electricity as a by-product was considered to be 70 $/MW-h [20]. 

In terms of techno-economic analysis, we determined breakeven prices. The breakeven selling prices for electricity and fuel ethanol as final products were calculated using Equations (2) and (3), respectively, as follows:

(2) $$\mathrm{BESP}=\frac{\sum_{i=1}^{n}C_{i}-\sum_{i=1}^{n}R_{i}}{Q}$$

(3) $$\mathrm{BFESP}=\frac{\sum_{i=1}^{n}C_{i}-\sum_{i=1}^{n}R_{i}}{Q}$$

where the BESP is the breakeven electricity-selling price ($/kW-h), BFESP is the breakeven fuel ethanol selling price ($/L), $C_{i}$ is the cost of *i*th step, $R_{i}$ is the revenue of *i*th by-product, and *Q* is the quantity of produced electricity (kW-h) or ethanol (L). 

### 2.2. Hatchery and Grow out Systems

The cultivation of seaweed consists of four stages [26]: (1) collection and settlement of zoospores on seed strings; (2) production of seedlings; (3) transplantation and outgrowing of seedlings; and (4) harvesting. The hatchery provides a protected area for young seedlings and facilities to establish grow out arrays before transferring to the main farm. Seaweeds can be cultivated in offshore/nearshore coastal farms as well as land-based ponds. Pond culture requires high investment and currently is used for specialty markets, and generally with integration and production of other aquatic products [8]. At present, nearshore farms are the most common, while offshore farming is often only experimental [14]. Offshore farming was considered in this study due to the potential of this method for large-scale farms [19]. Technical and economic data (capital, electricity, fuel, labor, consumables, etc.) of hatchery and grow out farm were taken from [19]. It was assumed that harvesting vessels and barges or boats to transfer harvested seaweed to the shore must be hired, similar to [19]. Thus the harvesting costs included the leasing cost of boats and barges, as well as labor and fuel consumption.

### 2.3. Drying Systems

The harvesting season, especially in cold regions with short growing seasons, is often too short. So the large volume of harvested seaweeds must be stored to continuously feed the ethanol process equipment. However, the high moisture content is an obstacle to safe and effective storage. Chemical treatments such as adding formalin or other additives (for fresh storage) have a negative effects on fermentation yields. Therefore, we assumed that the seaweed must be dried to achieve moisture content below 22% suitable for long-term storage [5]. On the other hand, dry material transportation is more efficient than wet, from both the energy and cost point of view. 

The heat energy needed to dry seaweed was obtained from Equation (4):

(4) $$H=\mathrm{WRHR}\times \left(MC_{i}-MC_{o}\right)$$

where H is the total heat required to dry one tonne of wet seaweed (MJ), WRHR is the seaweed water removal heat requirement (4000 MJ/t, [5]), $MC_{i}$ is the seaweed initial moisture content (85%, Kg/Kg), and $MC_{o}$, is the seaweed final moisture content (22%, Kg/Kg).

The costs of the drying operation, in addition to heat, were the costs of labor and the dryer facilities. The capital price of one typical 3-layer dryer with a thermal capacity of 1 t/h, was 60,000 $. This cost was converted to the cost at the desired scale using Equation (1).

### 2.4. Transportation Systems

To avoid additional costs and energy required to dry and transport the seaweed to the conversion system, the optimal situation would be to establish all drying, energy conversion, and by-product processing facilities integrated together, near the shorelines. It was assumed that the drying equipment is installed near the shore, so that the harvested seaweeds are delivered directly into the dryer equipment. Dried seaweed was then transferred by trucks to the conversion plants. Transportation costs for a 25 tonne truck were 2.6, 1.45, and 1.27 $/km for a 40, 160, and 320 km transportation radius, respectively [27]. The average distance of transportation between the dryer and final product conversion equipment was considered to be 40 km (25 miles). Also it was assumed that the labor demand for the transportation and drying steps were equal to the labor demand in the ethanol plant [25].

### 2.5. Conversion Systems

After delivery, two energy conversion methods were considered, as follows:

1. AD (anaerobic digestion) integrated with the CHP system: Biogas produced in an AD is burned in a CHP system to produce electricity. The waste product (digestate) from AD was used as fertilizer.
2. Ethanol production through fermentation: Ethanol is the main product in this method, and fermentation by-products are used as animal feed, digestate, or electricity production, based on the selected process method. Fermentation residuals can be converted into animal feed or can be digested to produce biogas and thus electricity. Specifically, the by-products in this method were animal feed or electricity and digestate as bio fertilizer, or the combination of these three products. According to [5], the rate of animal feed per liter of ethanol production is 1.21 kg. The amount of digestate production in residual fermentation followed by AD was equal to the amount of fresh seaweed fermentation in AD, but electricity production was reduced to 64% compared to scenario 1 (based on [10]).

#### 2.5.1. Fermentation

Potentially, the production of liquid biofuels from brown algae is high, due to the unique content of laminarin, mannitol, and alginate [8,16]. These structural polysaccharides and sugar alcohols should be broken down into their fundamental monomers before fermentation [14]. *Saccharomyces cerevisiae, Zymomonas mobilis*, glucanases, mannitol dehydrogenize, laminarinase, and cellulase are relatively common microorganisms and enzymes which are used for industrial fermentations [3,11,28,29,30]. To date, seaweed-based ethanol has been produced only on an experimental scale, so data for these processes must be estimated for large scale [5]. We assumed that the process of ethanol production from seaweeds may be similar to the process for corn ethanol conversion [5]. Therefore, with few exceptions, data of these processes, including energy and labor demand, equipment, by-products processing, and so on, were taken from [25]. 

#### 2.5.2. Anaerobic Digestion (AD)

Because of the typically low lignin content in macroalgae, it may be suitable for production of biogas in an anaerobic digester [10,14,31]. The overall conversion efficiency could be improved by integrating the methane production system with a CHP unit [10]. Therefore, it was assumed that the AD was integrated with a CHP unit. The inputs for anaerobic digestion, in addition to seaweed slurry (seaweed + water), included electricity (mainly for pumping) and heat (to heat the slurry from ambient temperature to the desired temperature). Electricity was supplied by the output electricity of gas engines. Recovered heat from the gas engine was more than the AD requirement [4,10]. However, because of the variability of AD requirements in different locations and seasons, it was assumed that all the produced heat was used to fulfill the AD requirement. Therefore the outputs of AD with CHP were electricity and digestate (as fertilizer). The economic data for AD and CHP were taken from [4] and [10]. Labor was assumed to be the same as for the ethanol plant [25].

### 2.6. Techno-Economic Analysis

All capital and operational costs were accounted for at multiple scales (up to 1.8 million tonnes of seaweed). The economic model was built using MS Excel, and six scenarios were examined using this spreadsheet—as depicted in Table 1. Breakeven sales prices for both electricity and ethanol were determined using this model, and will be discussed below.

## 3. Results and Discussion

### 3.1. Breakeven Price

Hatchery, drying and transportation methods were the same in all scenarios. However, the different combinations of cultivation methods (grid or longline), energy conversion methods (fermentation or AD) and by-product processing (animal feed, electricity, or digestate) created six different scenarios for analysis. Table 1 shows the breakeven price for the various scenarios in this study. The best result for ethanol resulted from ethanol produced via fermentation followed by anaerobic digestion of the residuals (1.55 ($/L), in scenario 3). However, the production costs in this scenario were partially compensated for by sales of the anaerobic digestion products (electricity and digestate); BFESP was about three times higher than 0.58 $/L in the study of [8]. One reason for this was the high cultivation costs (98 $/t dry) in the current study compared to that of [8] (25 ($/t dry)). Also the ethanol conversion rate in that study was very high (254 kg compared to 75 kg per one dry tonne of seaweed). The breakeven price of electricity produced via CHP was approximately 0.23 $/kW-h. This price is about 3-fold more than the electricity price in the market (0.07 $/kW-h) in the USA in 2013 [20], and it is 1.4 times more than 0.16 $/kW-h, which was obtained by [10]. Additionally, one of the most important issues which caused this difference was due to the different labor costs in the UK and USA. In addition to different cost components, the scale of production has a significant effect on the final product selling price. There was not much difference between grid and longline cultivation methods. However, the longline one is often preferred due to the higher productivity of this method (35 t/ha vs. 18 t/ha (wet basis)). 

**Figure 2:**
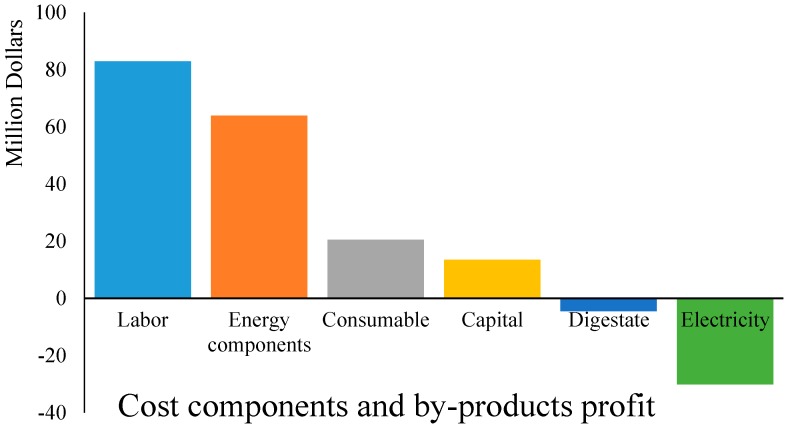
Separation of costs by major component for 95 ML ethanol production annually and anaerobic digestion of residuals. Negative costs actually mean profit (e.g., digestate and electricity).

Furthermore, Figure 2 shows the embedded cost components for scenario 3 for 95 ML ethanol production annually. It is clear in this figure that labor and energy (electricity, fuel, heat) are the most dominant cost components for ethanol production. And, as denoted by negative costs, sales of digestate and electricity actually are a result of product sales. Therefore, it appears that ethanol production from seaweed may be more cost efficient in the countries that have low energy prices and/or low labor cost. The share of labor is more prominent than energy, because firstly labor has the highest cost of all cost components, and secondly, part of the share of higher energy cost can be compensated for by the higher electricity selling price as a by-product. So it is recommended that energy conversion technologies and equipment could be established in countries such as China, Korea, and Indonesia where the labor cost is low but also seaweed cultivation experience is high. 

In all scenarios (Table 1), it was assumed that all residuals of fermentation could be used as animal feed or digest in AD to produce biogas. Another alternative could be that a portion of residuals be uses as animal feed while the rest could be used as digest for AD. Table 2 shows the BFESP when this approach would be implemented. BFESP in all cases was lower than 2 $/L. Because, the by-products produced in AD (electricity and fertilizer) are more valuable than animal feed, the BFESP decreases when the portion of fermentation residuals allocated to digest in AD increases. On the other hand, in situations when the value of animal feed compared to electricity and digestate increases, larger amounts could be allocated to animal feed.

### 3.2. Economic Analysis of the Production (Supply) Chain

**Figure 3:**
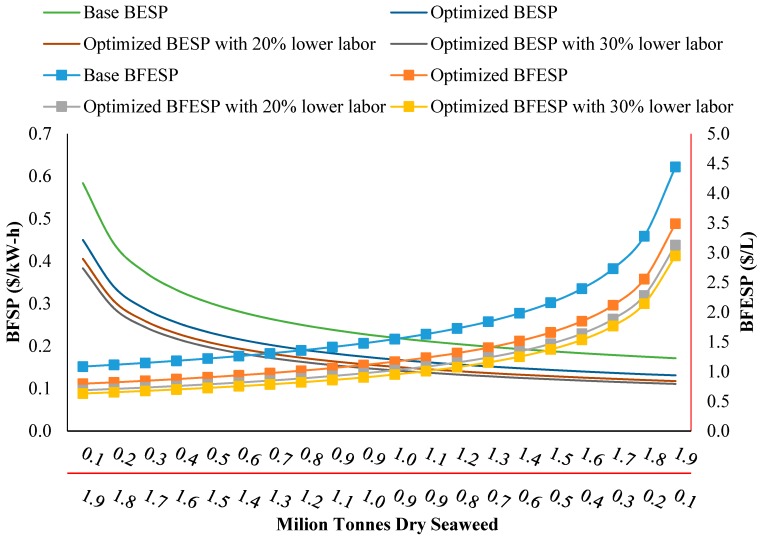
Changes in BESP (breakeven electricity selling price) ($/kW-h) and BFESP (breakeven fuel ethanol selling price, $/L, red axis) as a function of scale of seaweed production (million tonnes dry seaweed per year).

Some suggestions for economic optimization of the seaweed bioenergy supply chain are as follows:

1. Establish processing facilities and equipment in the closest location to the beach/water as possible; this will minimize the cost of transportation. Also, some of the seaweed can be consumed in fresh form in AD or fermentation (i.e., during the harvest season) without the need to dry and store the seaweed. Taking into account no transportation between the shoreline and the conversion equipment, and use of 25% of fresh seaweed, the BFESP and BESP can be reduced to approximately 1.17 ($/L) and 0.23 ($/kW-h), respectively.
2. Reduce production costs. As shown in Figure 2, the most dominant costs in the production chain are labor and energy inputs. So, with better management of cost components, the BESP and BFESP can be reduced. Considering the previous suggestion (establishment of integrated facilities near the shore) and by decreasing the labor cost by 20 and 30 percent, the BFESP can be decreased to 1.02 and 0.95 ($/L), respectively, and also BESP can be reduced to 0.16 ($/kW-h) and 0.15 ($/kW-h), respectively.
3. Increase productivity per unit area. The seaweed production yield in this study was only 5.25 and 2.7 (dry t/ha), respectively, for longline and grid farms; however, the average global yield of seaweed can range from 12 to 60 (dry t/ha) [17].
4. Extend the production scale. As shown in Figure 3, by increasing the production scale, costs can be pro-rated, and BESP and BFESP will be decreased.

### 3.3. Effect of Scale on Overall Cost

Figure 3 shows how these optimization procedures for BFESP in scenario 3 and BESP in scenario 5 (the lowest breakeven prices amongst all scenarios) (based on suggestions mentioned above), decline as a function of scale of seaweed production. It is clear in this figure that by increasing the production quantity, costs will exponentially decline, and BFESP and BESP will decrease. The required level to produce bioenergy from seaweed depends on the BFESP and BESP values. Currently, the marketing price of gasoline in the USA is 0.93 ($/L) [20]. To obtain this price level via seaweed, the annual production of seaweed must be 5.7 million tonnes (dry basis). If, for example, 20% of the cost could be subsidized by government policy (e.g., because of environmental benefits of seaweed derived bioethanol), the required level of production could be reduced to just 3.8 million tonnes (dry basis). This level can be further reduced by some of the approaches explained above. With regard to BFESP of 0.93 ($/L), the optimal level of production has been determined to be 1.44, 1.11 and 0.97 million tonnes annually for the optimized procedure, with 20% and 30% lower labor costs, respectively. Also by considering subsidized options, this level can be reduced to 1.0, 0.8, and 0.68 million tonnes for these options, respectively. 

The end use price of electricity for the industrial sector in the USA is approximately 0.07 ($/kW-h) [20,32]. To achieve this price level by seaweed, approximately 16.6 million tonnes (dry basis) seaweed must be used. If it is assumed that 20% of the cost can be subsidized by government policy, the optimal level will be reduced to 10.6 million tonnes (dry basis). And, by adapting various management solutions, to achieve BESP of 0.07 ($/kW-h), the required seaweed level will be reduced to 8.9, 6.6, and 5.7 million tonnes (dry basis) annually for the optimized procedures, with 20% and 30% lower labor costs, respectively. Also, by considering options with subsidies, this level can be reduced to 5.7, 4.3, and 3.7 million tonnes annually, respectively.

The economically feasible level of seaweed production to produce ethanol is much lower than that to produce electricity. The principal reason is that the management of by-products in ethanol production (at least as assumed in this study) resulted in higher economic values than for those in electricity production—it was assumed that residuals from ethanol production were digested to produce fertilizer and electricity, which were more valuable than animal feed.

## 4. Conclusions

Currently, the economical production of bioenergy from seaweed is not possible. However, by better management practices, such as reducing various cost components (especially labor) or improving the productivity in each stage of the seaweed supply chain, it may be possible to achieve a rational production cost. With the current situation, and applying the suggestions mentioned in this study for cost reductions, the minimum production of seaweed to have economically sustainable biofuel production was determined to be 680,000 dry tonnes annually. To have this quantity of production 129,500 ha needs to be cultivated. The cost of ethanol production at this scale was 0.93 ($/L). 

## Figures and Tables

**Table 1:** Scenarios analyzed in this study, and resulting in breakeven selling prices for the different scenarios.

Scenario	Farm Method	Conversion Method	By-Products		Final Product	Breakeven Selling Price of Final Product
1	Longline	Fermentation	Animal feed		Ethanol	1.87 ($/L)
2	Grid	Fermentation	Animal feed		Ethanol	1.93 ($/L)
3 *	**Longline**	**Fermentation**	**Electricity**	**Digestate**	**Ethanol**	**1.55 ($/L)**
4	Grid	Fermentation	Electricity	Digestate	Ethanol	1.61 ($/L)
5 *	**Longline**	**AD**	**Digestate**		**Electricity**	**0.23 ($/kW-h)**
6	Grid	AD	Digestate		Electricity	0.24 ($/kW-h)

* Bold indicates the lowest breakeven price for the scenarios.

**Table 2:** BFESP (breakeven fuel ethanol selling price), when the stated percentage of the residuals is used as animal feed *.

Percent of Residuals Used for Animal Food	100	90	80	70	60	50	40	30	20	10
BFESP ($/L)	1.87	1.85	1.83	1.81	1.79	1.76	1.74	1.71	1.68	1.65

* BFESP is the breakeven fuel ethanol selling price ($/L).
